# Preclinical Pharmacokinetic Assessment of a Promising Vasorelaxant, Analgesic, and Anti‐Inflammatory Prototype 5‐[1‐(4‐Fluorophenyl)‐1H‐pyrazol‐4‐yl]‐2H‐tetrazole (LQFM020) Through Selective Bioanalytical HPLC‐PDA‐Based Method

**DOI:** 10.1002/bmc.70082

**Published:** 2025-04-10

**Authors:** Lanussy Porfiro de Oliveira, Jerônimo Raimundo de Oliveira Neto, Thiago Sardinha de Oliveira, Lara Marques Naves, Stefanne Madalena Marques, Alessandro Carvalho Cruz, James Oluwagbamigbe Fajemiroye, Gustavo Pedrino, Luciano Morais Lião, Ricardo Menegatti, Luiz Carlos da Cunha

**Affiliations:** ^1^ Toxicopharmacological Studies and Research Core (NEPET‐UFG), College of Pharmacy Federal University of Goiás Goiânia Brazil; ^2^ Department of Pharmacy Federal University of Vales Do Jequitinhonha and Mucuri Diamantina Brazil; ^3^ Department of Physiology, Institute of Biological Sciences Federal University of Goiás Goiânia Brazil; ^4^ Nuclear Magnetic Resonance Laboratory, Institute of Chemistry Federal University of Goiás Goiânia Brazil; ^5^ Laboratory of Medicinal Pharmaceutical Chemistry, College of Pharmacy Federal University of Goiás Goiânia Brazil

**Keywords:** bioavailability, HPLC‐PDA, method validation, NSAID, preclinical pharmacokinetics

## Abstract

A simple and selective high‐performance liquid chromatography bioanalytical method was developed and validated to determine the pharmacokinetic parameters of 5‐[1‐(4‐fluorophenyl)‐1H‐pyrazol‐4‐yl]‐2H‐tetrazole (LQFM020) with promising vasorelaxant, anti‐inflammatory, and antinociceptive properties while verifying its potential hepatic enzyme induction or inhibition. Chromatographic separation was achieved using a reversed‐phase C18 column (ACE, 150 × 4.6 mm, 5 μm) with isocratic elution of a solvent mixture comprising acetonitrile and 0.2% formic acid (30:70, v/v). Detection of LQFM020 and the internal standard, piroxicam, was performed using a photodiode array detector. The method demonstrated excellent linearity (*r* > 0.998), with precision and accuracy within acceptable limits [intraday precision: 6.1%, interday precision: 9.3%; intraday accuracy: 113.2%, interday accuracy: 107.3%]. Pharmacokinetic studies revealed rapid oral absorption of LQFM020 at doses of 9, 18, and 36 mg/kg, as well as following a single intravenous dose (10 mg/kg). LQFM020 exhibited an absolute bioavailability of 46%, a relatively low apparent volume of distribution, and moderate elimination rates, suggesting extensive plasma protein binding. Additionally, LQFM020 showed no significant effect on the biotransformation of compounds mediated by the cytochrome P450 CYP3A4 enzyme. In conclusion, this new bioanalytical method supports preclinical studies and provides a basis for the utility of LQFM020 as a potential drug candidate.

## 1 Introduction

Nonsteroidal anti‐inflammatory drugs (NSAIDs) have been widely prescribed for decades to reduce pain and inflammation by inhibiting the cyclooxygenase enzyme (COX) and decreasing prostaglandin synthesis. However, NSAIDs elicit undesirable adverse reactions, especially hydro‐electrolyte renal disorders, leading to potential hypertension and cardiovascular disease rofecoxib (Minhas et al. 2023; Schmied 2023), besides gastrointestinal disturbs (Joo et al. 2020a) and bleeding (Joo et al. 2020b; Tsoupras et al. 2024).

**FIGURE 1:**
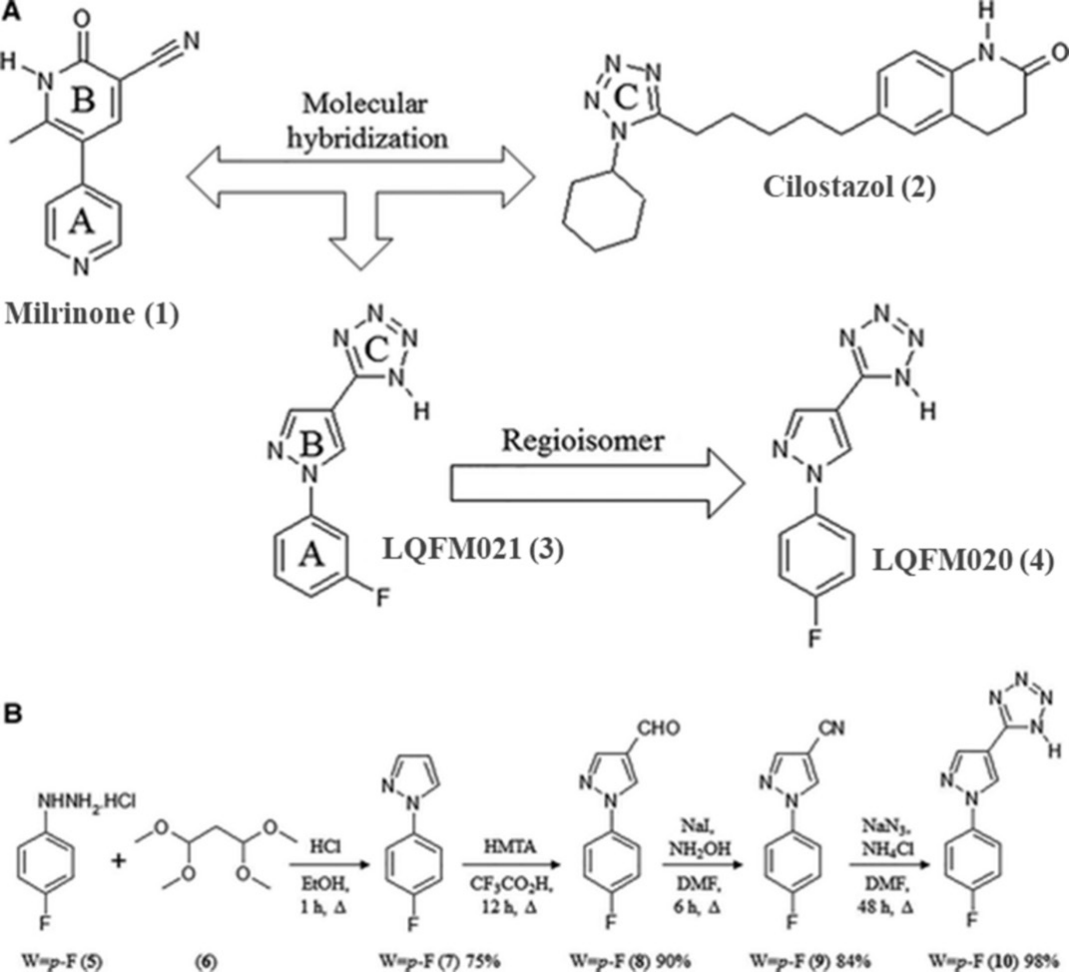
Structural design and synthetic route. (A) Structural design concept of new 5‐(1‐(4‐fluorophenyl)‐1H‐pyrazol‐4‐yl)‐1H‐tetrazole (4) and (B) synthetic route for the preparation of 5‐(1‐(4‐fluorophenyl)‐1H‐pyrazol‐4‐yl)‐1H‐tetrazole (4) (de Oliveira et al. 2017).

The synthesis, pharmacodynamic, and pharmacokinetic profiling of drug candidates are fundamental to the discovery of novel safe and effective NSAIDs. In this manner, 5‐[1‐(4‐fluorophenyl)‐1H‐pyrazol‐4‐yl]‐2H‐tetrazole (LQFM020) (4) was designed and synthesized from milrinone (1) and cilostazol (2) through a molecular modification as a drug prototype (Figure 1). The modulation of NO/cGMP signaling cascade and decrease of both defense cell migration and synthesis of pro‐inflammatory mediators were implicated in the excellent antinociceptive and anti‐inflammatory effects of LQFM020 prototype (Florentino et al. 2017). Also, LQFM020 has demonstrated an interesting vasorelaxant effect regarding the activity of K+ and Ca2+ channels, AC/cAMP, and GC/cGMP pathways (Ramos Martins et al. 2013), which might decrease the usual cardiovascular adverse effects of COX inhibitors. LQFM020 also showed a low toxicity, reducing 3T3 cell viability only at higher concentrations (0.35–1.4 mM), being well orally tolerated at doses ≤ 2000 mg/kg in mice (de Oliveira et al. 2017).

To date, the pharmacokinetic parameters of LQFM020 have not been explored, and due to its promising pharmacological effects, it is crucial to establish the behavior of this compound in the biological systems. Although molecular modifications are determinants to improve the selectivity and efficacy of potential drug candidates, it is primordial to achieve suitable absorption, distribution, metabolism, and excretion (ADME) parameters not to limit its early development stages. In addition to the description of the biological effect and pharmacokinetic profiling of a new potential drug candidate, the risk of drug–drug interactions, which seriously affect its safety and efficacy, demands further evaluation from LQFM020 prototype effects on enzymatic cytochrome P450 (CYP) complex. Drug‐related CYP inhibition or induction might alter plasma concentrations of drugs administered concomitantly, leading to undesired adverse effects or changes in their efficacy (Kato 2020).

Searching for simple, sensitive, and cheap methods that reliably determine compounds in complex matrixes remains challenging. Although several techniques might be available, high‐performance liquid chromatography (HPLC) and its various approaches keep demonstrating to be a good solution, even coupled with simple and cheap ultraviolet/photodiode array detection (UV/PDA), which might apply to the hundred biological samples as required by the pharmacokinetic studies (Tuzimski and Petruczynik 2020).

Therefore, this study aims to develop and validate a new, simple, and sensitive bioanalytical method to determine plasmatic LQFM020 using the HPLC‐PDA technique after oral and intravenous administration in rats. Moreover, this study sought to investigate the effect of LQFM020 on the CYP3A4 enzyme induction/inhibition and in silico prediction of LQFM020 physicochemical properties.

## 2 Material and Methods

### 2.1 Chemical Reagents

Acetonitrile and ethyl acetate HPLC grade were supplied by J. T. Baker (Phillipsburg, NJ), hydrochloric acid (fuming 37%) and formic acid were obtained from Vetec (Rio de Janeiro, Brazil), piroxicam (internal standard [IS]) was purchased from Sigma Chemical Co. (St. Louis, MO, USA), and 5‐[1‐(3‐fluorophenyl)‐1*H*‐pyrazol‐4‐yl]‐2*H*‐tetrazole (LQFM020, 98%) was synthetized by the Pharmaceutic and Medicinal Chemistry Laboratory (LQFM) from College of Pharmacy, Federal University of Goiás. All solutions were prepared using analytical‐grade reagents and double distilled water obtained from Gehaka Master A&D TOC system (São Paulo, Brazil). Urethane and isoflurane (2%–3% in O_2_) were purchased from Tanohalo (Cristália, SP, Brazil).

### 2.2 Instrumentation

An HPLC system (LC‐20AT model, Shimadzu Corporation, Kyoto, Japan) equipped with a low‐pressure quaternary gradient pump, column oven, autosampler, and PDA detector was used for this analysis. The separation was performed on a reversed‐phase C18 column (ACE, 150 × 4.6 mm i.d., 5 μm particle size) fitted with a security guard cartridge (4 × 3 mm) and maintained at 25°C ± 2°C. The mobile phase consisted of a mixture of acetonitrile and 0.2% formic acid (30:70, v/v) delivered in isocratic mode at a flow rate of 1.0 mL/min. An injection volume of 50 μL was used, with detection wavelengths set at 262 and 360 nm for LQFM020 and IS, respectively.

### 2.3 Calibration Standard and Quality Control Samples

LQFM020 and IS stock solution of 1 mg/mL were prepared in methanol, which were used to obtain working solutions in mobile phase. These was employed to prepare calibration curves, spiking blank rat plasma at concentration of 0.25, 0.5, 1.0, 2.5, 5.0, 10.0 and 20.0 μg/mL and quality controls at 0.25 μg/mL (limit of quantification [LOQ]), 0.75 μg/mL (low quality control [LQC]), 7.5 μg/mL (medium quality control [MQC]), 15.0 μg/mL (high quality control [HQC]), and 30.0 μg/mL (dilution quality control [DQC]).

### 2.4 Sample Preparation

Protein precipitation followed by a simple liquid–liquid extraction were applied to prepare the samples before chromatographic analysis using 125 μL of rat plasma spiked with 50 μL of IS, which was precipitated with 100 μL of HCl 1 mol/L. This mixture was vortexed for 30 s and centrifuged at 10000 rpm (4°C) for 5 min, and supernatant was transferred a clean tube. Then, 1 mL of ethyl acetate was added, vortexed for 1 min, and centrifuged at 10,000 rpm (4°C) for 5 min, and the upper organic layer was separated and evaporated under nitrogen flow. The residue was reconstituted with 100 μL of mobile phase and injected into HPLC‐PDA system.

### 2.5 Bioanalytical Method Validation

Bioanalytical method validation was conducted in accordance with the guideline of Brazilian Health Surveillance Agency (ANVISA) RDC 27/2012, analyzing several parameters, such as matrix effect, recovery, selectivity, linearity, precision and accuracy, and stabilities (short‐term, long‐term, and freezing/thawing cycles) (ANVISA 2012).

### 2.6 In Silico Physicochemical Properties' Evaluation

*ChemAxon* software (https://chemicalize.com/app) was employed to predict physicochemical characteristics of LQFM020 using the “rule of 5” proposed by Lipinski et al. (2001), which assesses following parameters: up to five hydrogen donor bonds; up to 10 hydrogen acceptor bonds; molecular weight less than 500 g/mol and cLogP less than 5. These rules are directed to define whether a substance has a good degree of absorption after oral administration.

### 2.7 CYP3A Enzyme Inhibition In Vitro Analysis

LQFM020 was evaluated for its ability to inhibit CYP3A4—the main subfamily responsible for drug metabolism in humans. The assay was conducted using a clean tube added with 100 μL of midazolam 10 μg/mL (CYP3A4 substrate), 100 μL of both monobasic potassium phosphate 1 M (as negative control), ketoconazole 20 μg/mL (as positive control), LQFM020 100 μg/mL (sample), and 100 μL of CYP3A4 enzyme solution 20 μg/mL, containing NADPH preincubate. Cold methanol was added after stirring these mixtures at 37°C for 2 h to stop the reaction and then centrifuged at 13,000 g for 10 min. The supernatant was filtered in Millex 0.22 μm pore and injected into HPLC system for the quantitation of midazolam. All samples were prepared in quintuplicate and analyzed by a HPLC‐PDA‐based method rigorously validated in our laboratory routine for the midazolam metabolites' quantification 1‐hydroxydazolam (1‐HMDZ) and 4‐hydroxydazolam (4‐HMDZ) (Ramirez De Oliveira et al. 2015).

### 2.8 Pharmacokinetic Study

#### 2.8.1 Animals

Male Wistar rats (250–300 g) obtained from Central Vivarium of Federal University of Goiás (UFG) were housed under a 12‐h light/dark cycle (22°C–23°C), with free access to food and water. This in vivo pharmacokinetic study was conducted in accordance with Ethical Principles in Animal Research, adopted by International Guiding Principles for Biomedical Research Involving Animals and approved by the Institutional Ethics in Research Committee at the UFG (Protocol CEUA/UFG 085/17). All efforts were made to minimize animal suffering as preconized by regulatory institutions.

#### 2.8.2 Sample Collection

The animals were anesthetized with isoflurane inhalation (2%–3% in O_2_; Tanohalo, Cristália, SP, Brazil) and underwent surgical procedure for the implantation of abdominal aorta cannula through right femoral artery and inferior cava vein to access rat bloodstream. LQFM020 was prepared in physiological solution containing DMSO 1% at dose of 10 mg/kg for intravenous administration (i.v., bolus), and then, blood samples (0.5 mL) were collected at 0, 0.5, 1, 2, 3, 4, 5, 6, and 24 h in heparinized microtubes. Oral administration (p.o.) was performed at LQFM020 dose of 9, 18, or 36 mg/kg prepared in physiological solution with DMSO 1%. Blood samples (0.5 mL) were collected at 0, 0.5, 1, 2, 3, 4, 5, 6, and 24 h in heparinized microtubes. Plasma samples were obtained by blood centrifugation (3000 g, 10 min) and were stored at −20°C.

#### 2.8.3 Pharmacokinetics and Statistical Analysis

LQFM020 pharmacokinetic parameters were obtained by non‐compartmental model, including peak plasma concentration (C_max_) and time to reach it (T_max_), elimination half‐life (t_1/2_), elimination constant (Kel), total area under the curve of plasma concentration versus time (AUC_T_), apparent distribution volume (Vd), clearance (Cl), and mean residence time (MRT). These pharmacokinetic parameters were calculated by Phoenix Connect 1.3.1 software (Certara, Princeton, NJ, USA).

LQFM020 absolute oral bioavailability *F(%)* was obtained applying AUC_T_ and dose (D) from p.o. and i.v. administration, according to the equation:

$$F\left(\%\right)=\frac{\mathrm{AUC}\left(p.o.\right)}{\mathrm{AUC}\left(i.v.\right)}\times \frac{D\left(i.v.\right)}{D\left(p.o.\right)}\times 100$$

Statistical analysis was performed using GraphPad Prism Version 5.01 software, applying a significant level of *p* < 0.05.

## 3 Results and Discussion

### 3.1 Method Development

Chromatographic parameters were optimized using previous physical–chemical and scientific publication data on analyte (LQFM020) and IS (piroxicam) to achieve better symmetry, sharpening, and resolution between chromatographic peaks and plasma interferents. Several tests were performed with different mobile and stationary phases using both gradient and isocratic elution mode, achieving best results on ACE C18 column (150 × 4.6 mm i.d., 5 μm particle size), eluted with a simple mixture of acetonitrile/formic acid 0.2% (30:70, v/v), flowing an isocratic elution of 1.0 mL/min. LQFM020 and IS retention times of 5.8 and 8.1, respectively, were considered adequate and relatively better than some HPLC‐UV/PDA or RF (fluorescence detector)‐based bioanalytical methods to determine main NSAID and its potential drug candidates (Han et al. 2019; Pavan Kumar et al. 2006; Zhang et al. 2003). In fact, to quantify valdecoxib and its main metabolite (M1), Zhang et al. (2003) obtained a total chromatographic run of 30 min, which is, to some extent, incompatible with hundreds to thousands of samples usually analyzed in pharmacokinetic studies (Zhang et al. 2003). The same impact was shown in a HPLC‐FL (spectrofluorimetric detection)‐based bioanalytical method, developed to determine a novel COX‐2 inhibitor cimicoxib, achieving a total chromatographic run time of 15 min. It is evident that many LC‐MS/MS‐based methods (HPLC or UHPLC) were developed for the NSAID determination with shortest analytical run, but these techniques not always are available, especially because of its costly and maintenance (Starek 2011).

In this study, preliminary results from only protein precipitation of plasma samples, using acetonitrile and methanol, showed several interferents. Then, to work around these issues, an LLE was applied to, using different organic solvents such as dichloromethane, ethyl acetate, methyl tert‐butyl ether, or solvent mixtures, including acid and basic pH changes. The best resolution and recovery results for both LQFM020 (86.5% ± 2.6%) and IS (85.8% ± 1.1%) associated with better sample clean‐up (no interference) were achieved using a previous protein precipitation (100 μL HCl 1 mol/L) followed by LLE adding 1 mL of ethyl acetate.

### 3.2 Method Validation

#### 3.2.1 Selectivity

**FIGURE 2:**
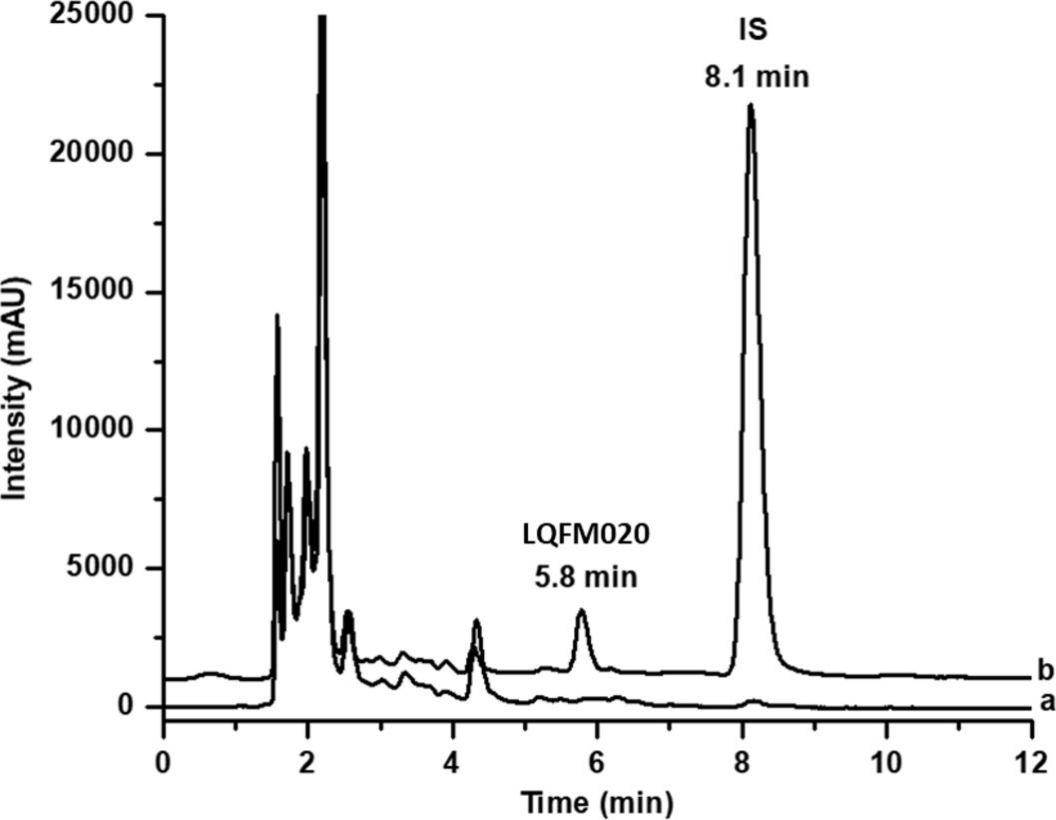
Representative chromatogram of blank plasma sample (a) and LQFM020 at 0.25 μg/mL and IS (piroxicam at 10 μg/mL) (b). The wavelength was set at 262 nm.

The selectivity of this bioanalytical method was performed by comparing chromatograms between blank plasma (including normal, lipemic, and hemolyzed samples) and LOQ of LQFM020 and IS extracted plasma samples. All results demonstrated there were no interferents from blank plasma at retention times of both analyte and IS, as depicted in representative chromatograms Figure 2.

#### 3.2.2 Carryover Effect

The injections of high calibration curve concentration sample (20.0 μg/mL) spiked with IS (10.0 μg/mL) followed by a blank plasma sample were used to evaluate carryover effect. No peak was detected at the retention time of analyte and IS in blank plasma sample, demonstrating no column or HPLC system adherence and an autosampler cleaning efficiency (data not shown).

#### 3.2.3 Calibration Curves, Precision, and Accuracy

A good linearity was obtained over the LQFM020 concentration ranging from 0.25 to 20.0 μg/mL, demonstrated by an excellent correlation coefficient (*r*) > 0.998, which was represented by a linear regression equation y = 0.022 + 0.117x. Precision and accuracy assays showed adequate results with maximum coefficient of variation (CV) of 6.1% (intraday) and 9.3% (interday) and relative error (RE) ranging from 88.3% to 113.2% for intraday and from 99.4% to 107.3% for interday, respectively (Table 1). These acceptable values are in accordance with recommended by regulatory agency and guidelines for bioanalytical methods (ANVISA 2012).

**TABLE 1:** Intra‐ and interday precision and accuracy data for the quantification of LQFM020 in rat plasma.

[Nominal] μg/mL	[Intraday mean] (*n* = 6)	Intraday		[Interday mean] (*n* = 6)	Interday	
		Precision (CV%)	Accuracy (RE%)		Precision (CV%)	Accuracy (RE%)
LOQ 0.25	0.22	5.6	88.3	0.25	9.3	100.7
	0.26	2.3	105.8			
	0.25	1.7	107.0			
LQC 0.75	0.73	2.9	96.7	0.74	3.1	99.2
	0.77	1.8	102.2			
	0.74	1.2	98.0			
MQC 7.5	8.02	4.7	106.9	7.98	4.3	106.4
	7.95	1.7	106.0			
	7.96	6.1	106.2			
HQC 15.0	16.98	5.5	113.2	16.14	6.0	107.3
	16.35	2.4	109.0			
	15.10	2.4	100.7			
DQC 30.0	30.57	1.7	101.9	29.77	5.0	99.4
	30.84	2.6	102.8			
	27.90	2.6	93.0			

Abbreviations: CV = coefficient of variation, DQC = dilution quality control, HQC = high quality control, LOQ = limit of quantification, LQC = low quality control, MQC = medium quality control, RE = relative error.

#### 3.2.4 Stability

LQFM020 has been demonstrated stable at the storage and handling conditions, according to the experimental results achieved in three different stabilities assays. Indeed, rat plasma concentrations (*n* = 3) of LQC and HQC presented a nonsignificant degradation below ±15% (RE), ranging from 89.2 ± 2.2% (short term) to 107.5 ± 1.8% (long term) at the end of storage condition times, as shown in Table 2.

**TABLE 2:** Stability of LQFM020 in different conditions in rat plasma.

Stability	[Nominal] μg/mL	[Experimental] μg/mL	Accuracy (RE)
			(Mean ± CV%)
Short duration (RT, 4 h)	0.75	0.67	89.2 ± 2.2
	15	14.17	94.5 ± 5.2
Freeze–thaw (3 cycles)	0.75	0.72	96.4 ± 2.6
	15	14.40	96.0 ± 5.3
Long duration (30 days, −20°C)	0.75	0.77	102.1 ± 6.5
	15	16.12	107.5 ± 1.8

Abbreviations: CV = coefficient of variation, RT = room temperature.

### 3.3 In Silico Prediction of LQFM020 Physicochemical Properties

According to the main basic physicochemical properties, such as molecular weight of 230.206 g/mol, polar surface area (PSA) of 72.28 Å and coefficient partition o/w (logP) = 1.59, LQFM020 might demonstrate a good in vivo oral absorption. In fact, as report by Ertl et al., a compound might be considered almost fully absorbed presenting a PSA < 60 Å and logP between 1.35 and 1.80 (for intestinal absorption), but with PSA > 140 Å and logP < 1.0, less than 10% probable will be absorbed (Ertl et al. 2000; Österberg and Norinder 2000). Moreover, LQFM020 achieved a logS −2.67, and considering most current oral drugs have a logS ranging −1 to −5, it might be suggested that LQFM020 shows suitable aqueous solubility and lipophilicity to cross the biological membranes (Lipinski et al. 2001). Consequently, it seems that LQFM020 might has a high permeability, as it complies with Lipinski's “rule of 5”, which predicts a cLogP < 5, a molecular weight less than 500 g/mol and hydrogen bond donors not more than 5, followed by hydrogen bond acceptors less than 10 (Table 3) (Lu et al. 2004).

**TABLE 3:** In silico prediction of LQFM020 physicochemical properties.

Estimated parameter	Result
Molecular weight	230.206 g/mol
Polar surface area	72.28 Å
Acceptor H^+^	3
Donor H^+^	1
LogP	1.59
LogD	2.32
LogS	−2.67
pKa	4.20

### 3.4 Inhibition of CYP3A4 Enzymes by LQFM020

**FIGURE 3:**
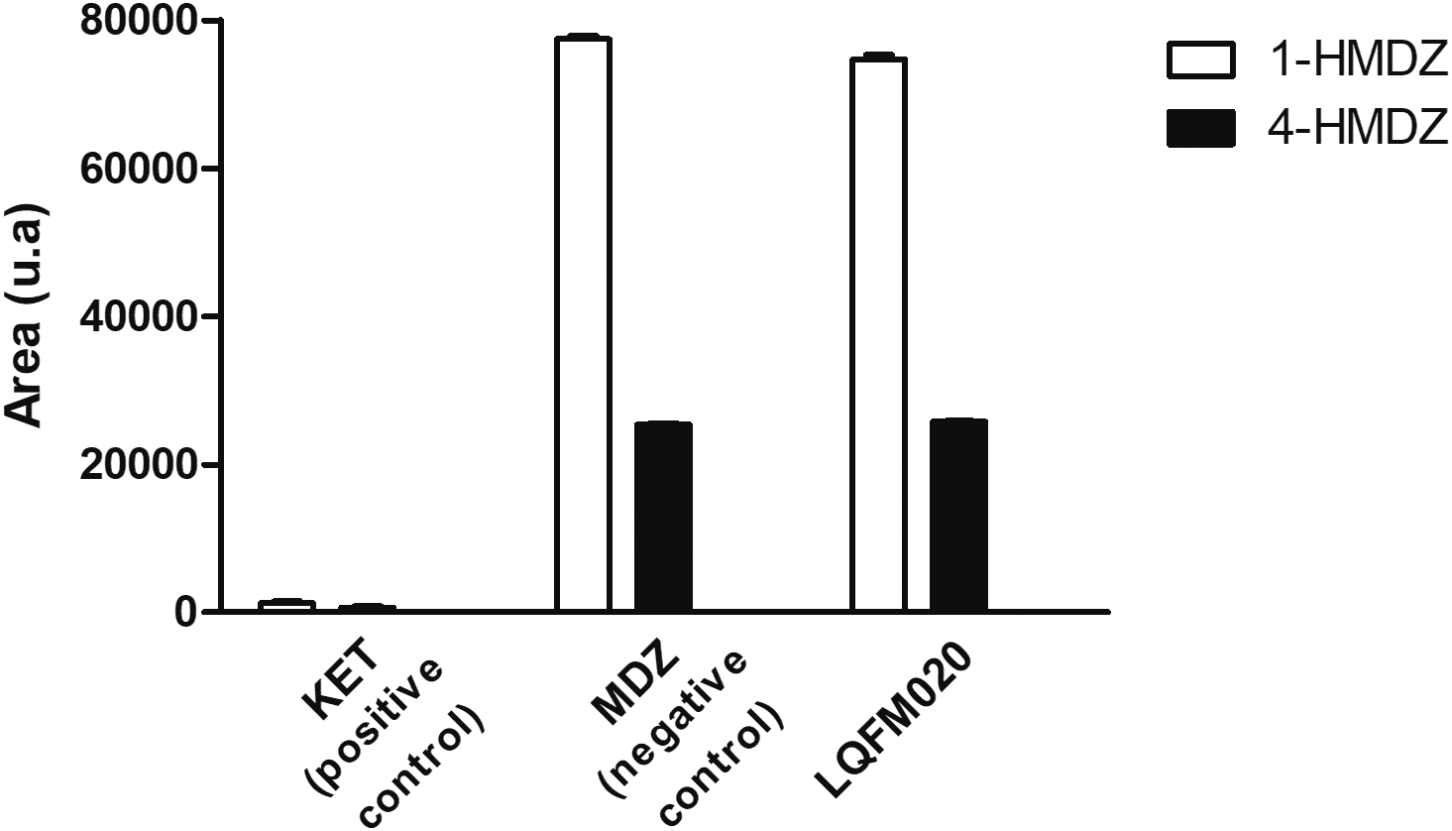
In vitro biotransformation assay to evaluate LQFM020 on CYP3A4 activity. KET (positive control) represents a medium composed of CYP3A4 enzyme added of midazolam plus ketoconazole (enzyme inhibitor); buffer (negative control) is the CYP3A4 enzyme medium added of midazolam (enzyme substrate); and LQFM020 includes CYP3A4 enzyme medium added to a test compound plus enzyme substrate (midazolam). 1‐HMDZ, 1‐hydroxydazolam; 4‐HMDZ, 4‐hydroxydazolam.

Several NSAIDs, such as pyrazoles derivatives (phenylbutazone and celecoxib), might undergo hepatic biotransformation, and potentially inhibit enzymes from liver microsomal system (Davies et al. 2000). Because of this, LQFM020 was evaluated for inhibition of the main enzyme of cytochrome P50 CYP3A4. The results demonstrated an absence of inhibition, as the same peak areas of the main biomarker metabolites 4‐hydroxydazolam (4‐HMDZ) and 1‐hydroxydazolam (1‐HMDZ) from midazolam added with LQFM020 were achieved, compared to the sample without any inhibitor (negative control). Conversely, a significant inhibition was demonstrated for ketoconazole (positive control), which blocked CYP3A4 activity on midazolam biotransformation, decreasing significantly the peak areas of 4‐HMDZ and 1‐HMDZ metabolites (*p* < 0.05), as depicted in Figure 3. These findings highlight an important characteristic of LQFM020, as CYP3A4 is responsible for the several drug biotransformation and frequently involved in drug interactions (Zhou et al. 2007).

### 3.5 Pharmacokinetic Study

This bioanalytical HPLC‐PDA‐based method showed selectivity, linearity, precision, and accuracy suitable to apply successfully to the pharmacokinetic study to quantify the drug candidate LQFM020 in rat plasma, after three different doses administered orally and a single dose administered intravenously. More than 400 plasma samples were analyzed, including calibration curves, QC, and rat plasma samples, achieving high‐throughput essential for pharmacokinetic studies that require many samples analysis (Xu et al. 2007).

Apparently, according to the rat plasma concentrations after oral administration of 9, 18, or 36 mg/kg doses, LQFM020 was rapidly absorbed from gastrointestinal tract, reaching maximum concentrations (Cmax) of 16.59, 26.57, and 55.25 μg/mL, at the time (Tmax) of 1.66, 0.83, and 1.67 h, respectively, as shown in Table 4. In fact, LQFM020 demonstrated a relatively good extent absorption after to be exposure to the rat organism as described by high total AUC values, ranging from 69.77 to 286.70 h.μg/mL, after oral 9 and 36 mg/kg doses, respectively.

**TABLE 4:** Pharmacokinetic parameters of LQFM020 after oral administration doses of 9, 18, or 36 mg/kg in male adult rats (mean ± SEM, *n* = 3).

Dose (mg/kg)	t_1/2_ (h)	C_max_ (μg/mL)	T_max_ (h)	AUC_T_ (h.μg/mL)	Vd/F (L/kg)	Cl_T_ (mL/min/kg)
9	5.82 ± 2.35	16.59 ± 4.41	1.66 ± 0.66	69.77 ± 5.33	0.86 ± 0.40	0.11 ± 0.01
18	5.85 ± 0.96	26.57 ± 2.03	0.83 ± 0.17	109.65 ± 17.11	1.44 ± 0.36	0.16 ± 0.02
36	6.74 ± 2.47	55.25 ± 21.08	1.67 ± 0.66	286.70 ± 82.38	1.16 ± 0.26	0.15 ± 0.06

Abbreviations: ASC_T_ = area under the concentration curve versus time 0 to infinity (total), Cl_T_ = total clearance, C_max_ = maximum concentration, Kel = elimination constant, t_1/2_ = elimination half‐life, T_max_ = time to reach maximum concentration, V_d_ = volume of distribution.

**FIGURE 4:**
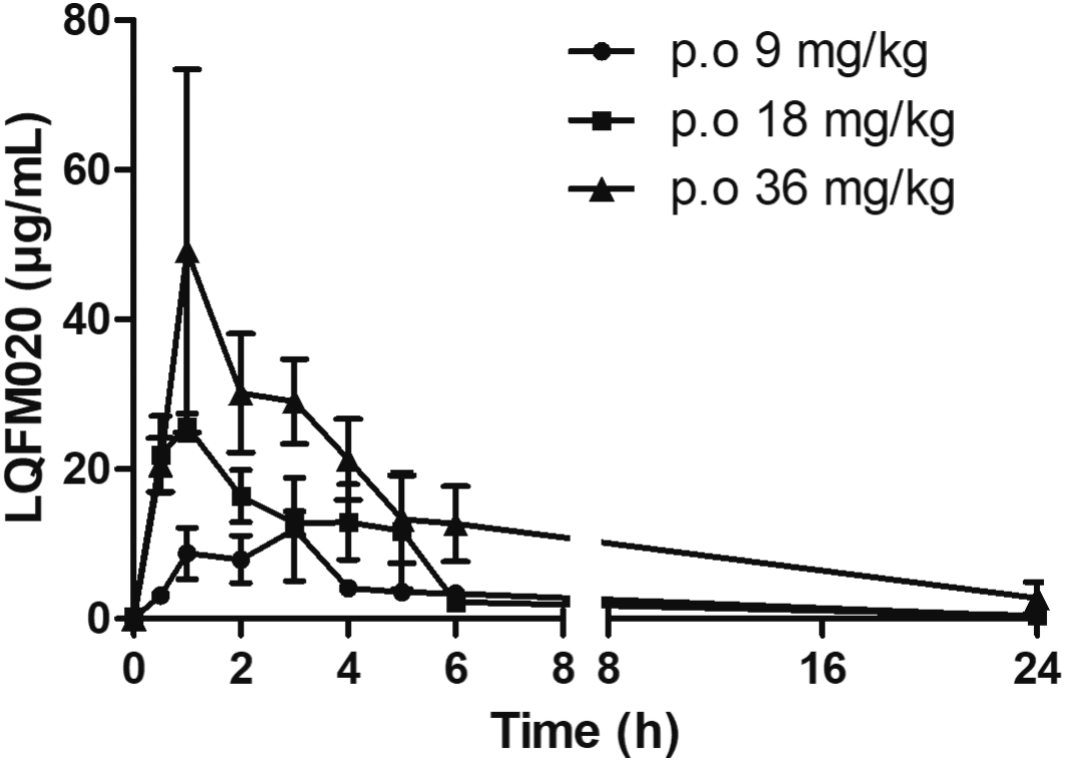
Pharmacokinetic profiles of LQFM020 following oral administration at 9, 18, or 36 mg/kg doses in male adult rats (*n* = 3).

Interestingly, it might be suggested that LQFM020 has first‐order kinetics in rats, due to the relative proportionality between oral doses administered and plasma concentrations obtained, evidenced by both Cmax and AUC_T_ values (Figure 4). Overall, these results are aligned with main LQFM020 physicochemical properties, especially on Lipinski's “rule of 5” previously determined in this study. Most current NSAIDs used clinically are easily absorbed through the gastrointestinal tract, achieving maximum concentrations within 1–4 h. Indeed, for example, the pyrazole derivative celecoxib (COX‐2 selective widely used nowadays) has a moderate to high absorption rate, similar to LQFM020 administered orally, reaching a Tmax within 2–4 h (Davies et al. 2000; Dhondt et al. 2017; Gong et al. 2012).

The solubility and permeability of an oral drug in the gastrointestinal tract fluids are key factors for its absorption and suitable therapeutic activity, influencing significantly its bioavailability (Fan and de Lannoy 2014; Martinez and Amidon 2002). In this study, it was possible to determine the LQFM020 absolute oral bioavailability (*F*(%)) in rat using AUC and dose from intravenous and oral administration. The result indicated that 46% of an oral LQFM020 dose reached the systemic circulation, which is similar to some selective COX‐2 inhibitors, ranging from 20% to 40%, especially due to its low solubility in aqueous medium (Dhondt et al. 2017; Shi and Klotz 2008). In addition, another factor to explain relatively low oral bioavailability of a drug is the pre‐systemic elimination, and apparently, LQFM020 might suffer a significant hepatic first‐pass metabolism. Therefore, preclinical pharmacokinetic studies are essential to investigate the release of potential drug candidates with systemic action through the intestinal membrane and to develop strategies to improve its oral absorption and to understand the impact on its pharmacological effect (Fan and de Lannoy 2014).

**FIGURE 5:**
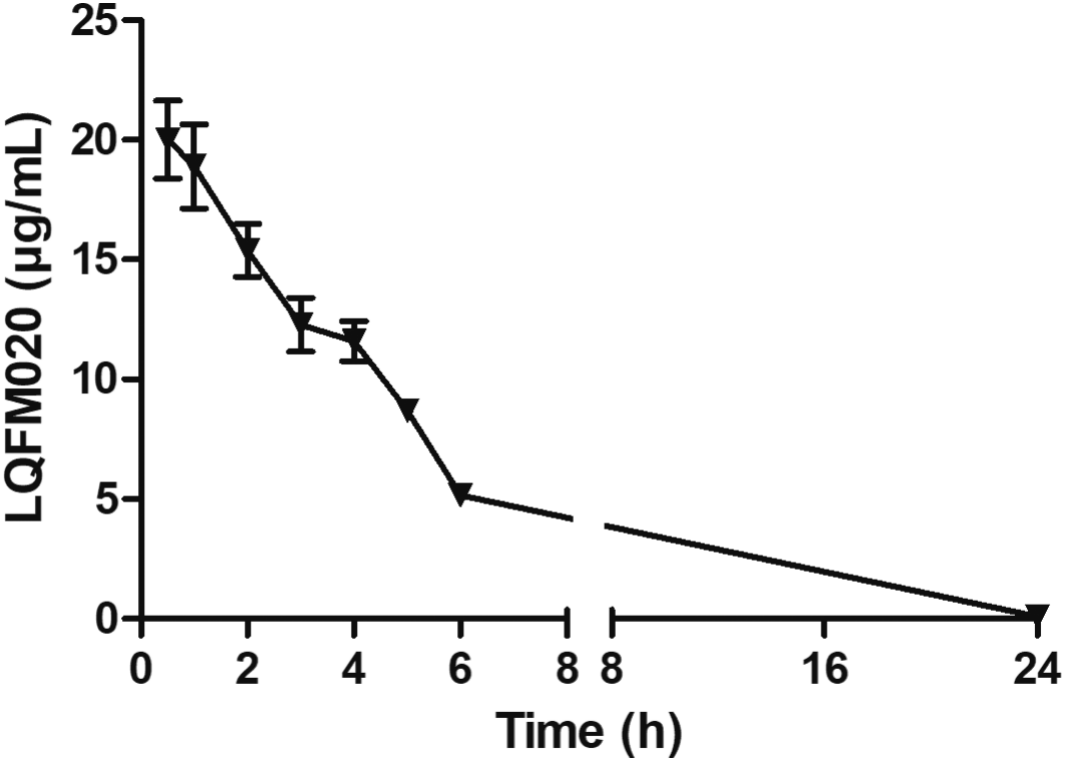
Pharmacokinetic profile of LQFM020 following i.v. administration at 10 mg/kg dose in male adult rats (*n* = 3).

The plasma concentration–time profile observed after a single IV bolus LQFM020 administration was shown in Figure 5, and the non‐compartmental pharmacokinetic parameters were depicted in Table 5.

**TABLE 5:** Pharmacokinetic parameters of LQFM020 following i.v. administration of 10 mg/kg dose in male adult rats (*n* = 3).

Animal	t_1/2_ (h)	AUC_T_ (h*μg/mL)	Vd/F (L/kg)	Cl_T_/F (mL/min/kg)
1	2.83	1228.31	0.032	0.008
2	3.06	1064.89	0.041	0.009
3	3.26	1314.07	0.036	0.007
Mean ± SEM	3.05 ± 0.12	1220.54 ± 77.87	0.03 ± 0.00	0.008 ± 0.00

Abbreviations: AUC_T_ = total area under curve of plasma concentration versus time, Kel = elimination constant, Vd/F = apparent volume of distribution, Cl_T_/F = total clearance, t_1/2_ = plasma elimination half‐life.

The results of LQFM020 rat plasma concentrations from intravenous administration indicated a low variability between animals, achieving a good consistency of main pharmacokinetic parameters. As demonstrated for oral administration, LQFM020 plasma concentrations declined slowly at the elimination phase, achieving a moderate‐to‐high elimination half‐time (t_1/2_) of approximately 6 and 3 h for oral and intravenous administration, respectively. These results might indicate an LQFM020 high plasma protein binding, agreeing with a relatively low apparent volume of distribution (0.03 L/kg, I.V.) in rats determined in this study. In fact, the lower the distribution volume of a drug, the worse its distribution to the organism tissues, increasing the bloodstream residence time. It might be explained because LQFM020 has a pKa of 4.20 (weak acid) as demonstrated by in silico analyses, promoting its ionization at the neutral blood pH and consequently decreasing possible crosses through tissues lipidic membrane of rat organs. Corroborating with these inferences, LQFM020 also demonstrated a relatively low elimination rate (Cl_T_ = 0.008 mL/min/kg, I.V.), because apparent Vd/F is directly proportional to plasma depuration and conversely proportional to the high LQFM020 AUCT of 1220.54 h.μg/mL (Table 5). The relative slow depuration rate of LQFM020 might also indicate its extensive reabsorption in the nephron, due to the lower pH values in rodents, promoting its non‐ionized form easier permeable through renal tubes and back to the rat bloodstream (Fan and de Lannoy 2014; Tannehill‐Gregg et al. 2009).

Finally, this is the first in vivo preclinical experiment designed to understand the pharmacokinetics of promising potential NSAID candidate LQFM020 in rats. The results obtained might support the next steps in drug development using a simple, sensitive, accurate, and precise bioanalytical HPLC‐PDA‐based method developed and validated in this study.

## 4 Conclusion

In this work, a simple and sensitive bioanalytical HPLC‐PDA‐based method was developed and validated for the determination of promising vasorelaxant, analgesic, and anti‐inflammatory 5‐[1‐(4‐fluorophenyl)‐1h‐pyrazol‐4‐yl]‐2h‐tetrazole (LQFM020) in rat plasma samples. According to the suitable precision, accuracy, and linearity achieved on validation procedures, this method was successfully applied to the first preclinical LQFM020 pharmacokinetic study through oral and intravenous administration. Therefore, an LQFM020 absolute bioavailability of 46% was determined, indicating a good absorption, especially compared to most NSAIDs used in clinical currently (ranging from 20% to 40%), besides a relatively low apparent volume of distribution and elimination rates, which might suggest an extensive plasma protein binding. Moreover, the results from the enzymatic inhibition assay indicated that LQFM020 does not change the biotransformation of compounds by cytochrome P450 CYP3A4 enzyme. Altogether, this new bioanalytical method will support preclinical and clinical future studies to evaluate the promising effects and behavior of LQFM020 potential drug candidates.

## Author Contributions

Lanussy Porfiro de Oliveira, Thiago Sardinha de Oliveira, Jerônimo Raimundo de Oliveira Neto, and Alessandro de Carvalho Cruz were responsible for acquisition, analysis and interpretation of data, and preparation and revision of manuscript. Ricardo Menegatti was responsible to the synthesis of new compound and revision of manuscript. Luciano Morais Lião was responsible for confirming the structure of LQFM020 and revision of manuscript. Lara Marques Neves, Stefanne Madalena Marques, and Gustavo Pedrino were responsible for experimental procedures of cannulation of the animals and blood collect. James Oluwagbamigbe Fajemiroye and Luiz Carlos da Cunha were responsible for concept, design, and preparation of the manuscript. All authors read and approved the final version of the manuscript.

## Conflicts of Interest

The authors declare no conflicts of interest.
